# Learning in lockdown: Using the COVID‐19 crisis to teach children about food and climate change

**DOI:** 10.1111/nbu.12489

**Published:** 2021-02-26

**Authors:** A. Kluczkovski, R. Lait, C. A. Martins, C. Reynolds, P. Smith, Z. Woffenden, J. Lynch, A. Frankowska, F. Harris, D. Johnson, J. C. G. Halford, J. Cook, J. Tereza da Silva, X. Schmidt Rivera, J. L. Huppert, M. Lord, J. Mclaughlin, S. Bridle

**Affiliations:** ^1^ The University of Manchester Manchester UK; ^2^ Centre for Food Policy City, University of London London UK; ^3^ University of Aberdeen Aberdeen UK; ^4^ Fairway Primary School Manchester UK; ^5^ University of Oxford Oxford UK; ^6^ Centre on Climate Change and Planetary Health London School of Hygiene and Tropical Medicine London UK; ^7^ Department of Earth and Environmental Sciences The University of Manchester Manchester UK; ^8^ University of Leeds Leeds UK; ^9^ Department of Environment and Geography The University of York York UK; ^10^ HCor Research Institute São Paulo Brazil; ^11^ Equitable Development and Resilience Research Group (EDR), Centre for Sustainable Energy use in Food chains (CSEF), College of Engineering, Design and Physical Sciences Brunel University London Uxbridge UK; ^12^ Jesus College King’s College London UK; ^13^ Ogden Trust Regional Rep Manchester UK

**Keywords:** climate change, education, food

## Abstract

Food systems are significant sources of global greenhouse gas emissions (GHGE). Since emission intensity varies greatly between different foods, changing food choices towards those with lower GHGE could make an important contribution to mitigating climate change. Public engagement events offer an opportunity to communicate these multifaceted issues and raise awareness about the climate change impact of food choices. An interdisciplinary team of researchers was preparing food and climate change educational activities for summer 2020. However, the COVID‐19 pandemic and lockdown disrupted these plans. In this paper, we report on shifting these events online over the month of June 2020. We discuss what we did and the reception to our online programme. We then reflect on and highlight issues that arose. These relate to: (1) the power dynamics of children, diet and climate change; (2) mental health, diet and COVID‐19; (3) engaging the wider science, agriculture and food communities; (4) the benefits of being unfunded and the homemade nature of this programme; (5) the food system, STEAM (science, technology, engineering, arts and mathematics) and diversity; and (6) how our work fits into our ongoing journey of food and climate change education.

## INTRODUCTION

Food systems are important sources of global greenhouse gas emissions (GHGE) (Mbow et al., 2019). Since emission intensity varies greatly between different foods, changing food choices towards those with lower GHGE could make an important contribution to mitigating climate change (Willett et al., 2019). Seventeen of the Sustainable Development Goals recognise climate change as one of the grand challenges that needs global action and commitment to mitigate and lessen the effects for future generations (Piscopo, 2015). Education and public engagement events offer an opportunity to communicate these multifaceted issues and raise awareness about the climate change impact of food choices.

In the summer of 2019, the *Take a Bite Out of Climate Change* *(TakeABiteCC)* outreach activities were run at various science festivals around the UK (Kluczkovski et al., 2020), as well as adapted for use in workshops in India, Brazil and Myanmar. *TakeABiteCC* was organised to share widely the scientific evidence about how food and agriculture contribute to climate change, providing easily accessible information and fun activities to help citizens understand how they can help reduce their climatic impact. Preparations were underway to develop *TakeABiteCC* activities for summer 2020 including teaching resources for open days and schools. However, the COVID‐19 pandemic and lockdown disrupted these plans.

In March 2020, the *TakeABiteCC* team held an online meeting to discuss the impact of COVID‐19 and the resulting lockdown. We reviewed the facts that COVID‐19 and the resulting lockdown had: (1) dramatically changed people's food habits (Hubbub, 2020); (2) closed schools, leading to many schoolchildren experiencing distance/online learning for the first time; and (3) increased the time that people were at home, changing daily routines (including school work and families eating together). There appeared to be a ‘window of opportunity’ to make people aware of the important role of the food system in climate change. The *TakeABiteCC* team decided to develop free online materials on this subject that were released each weekday in the month of June 2020. Our main target audience was English speaking schoolchildren aged 7–14 years, along with school teachers and parents/carers.

This report reflects on what we did, and the issues we faced in adapting our food and climate change materials to online teaching. Overall, we found this to be a positive, community building experience. Sharing our experience in this report, we encourage other groups to use our materials and engage in similar activities with the wider community.

### Who we are

The *TakeABiteCC* team includes researchers from various disciplines from different UK and international universities. The diversity of researcher's backgrounds allowed us to cover a wide range of topics relating to food and climate.

### What we did

Under the banner *Take a Bite Out of Climate Change At Home* (#TakeABiteAtHome), the team produced new video content for every weekday of June 2020. Each day of the week featured a different type of video and activity worksheets were released weekly. These were embedded on our website (https://www. TakeABiteCC.org/athome.html) and YouTube channel created especially for this project (https://www.youtube.com/channel/UC1siBKLoy4bFXr8F6Y3Bpqg). The project was co‐ordinated with Slack communication software and Google Docs word processor tool.

We publicised the project by emailing promotional flyers to approximately 20 educational institutions among school mailing lists, school‐related partners and 10 press offices 2 weeks before the project started. The promotional flyers contained information regarding the schedule of events, the team and ways to sign up to emails to receive more information. The flyers were developed with appropriate language to target different audiences: a school‐specific one targeted at teachers (which included an option to see the content in advance for lesson planning), and another flyer tailored towards schoolchildren. The promotional flyers were also published on social media (Instagram, Facebook, Twitter, LinkedIn) through the team's accounts.

Each week had a theme related to a different stage of the food system: Week 1 (Planet to Plate), Week 2 (At the Farm), Week 3 (At the Shops), Week 4 (At Home) and a 2‐day Wrap Up. Each day of the week was themed to have different types of videos.

#### Go Mondays

A pre‐recorded 3‐minute video introducing the week's theme and communicating three learning outcomes and three facts in an engaging way released every Monday.

#### Work‐it‐out Tuesdays

**FIGURE 1:**
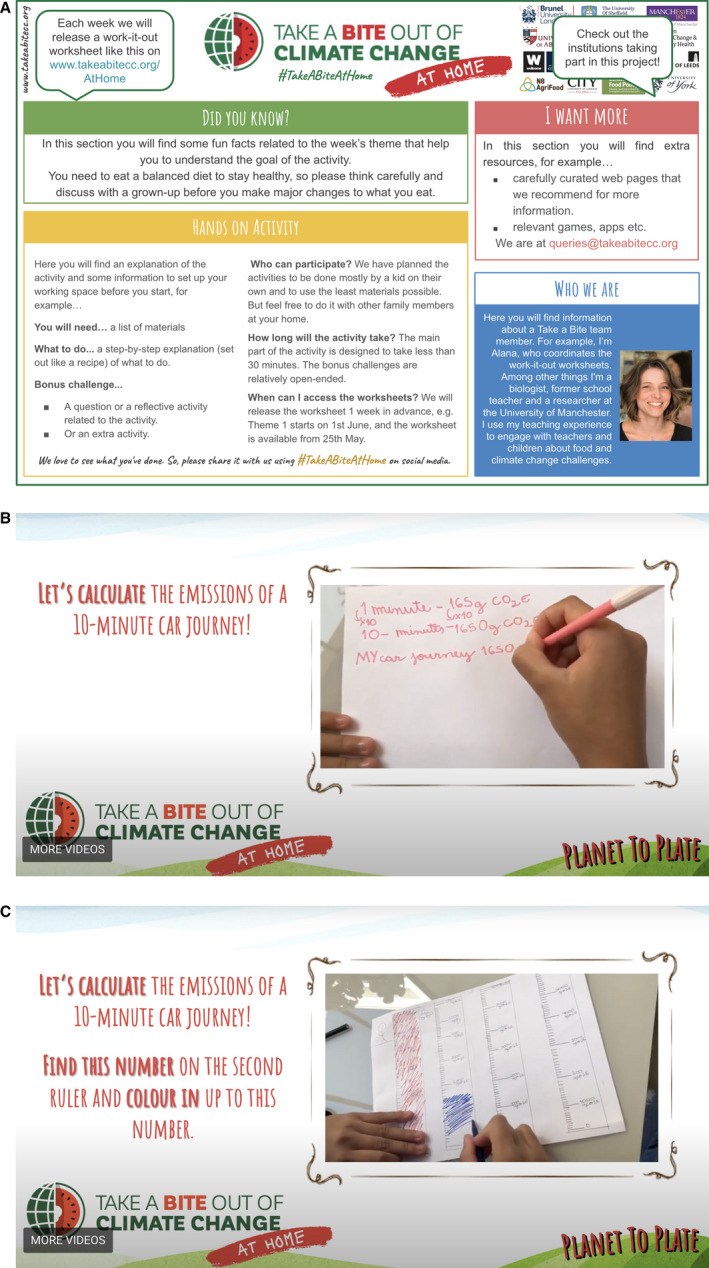
(a) An example of a Work‐it‐out Tuesdays’ activity sheet. (b,c) Screenshots from a worksheet walkthrough video showing a child carrying out calculations and illustrating them graphically. [Colour figure can be viewed at wileyonlinelibrary.com]

A 1‐page activity worksheet for schoolchildren to complete on their own, along with a supporting page of created materials (available 1 week in advance on the website, as requested by teachers to support lesson planning). We developed a template sheet that explained each section of the activity worksheet and how it would work every week (Figure 1a). We filmed walkthrough videos showing a child completing the hands‐on activities, to help children working without adult support, thus engaging a broader spectrum of learners (Figure 1b,c).

#### Interview Wednesdays

**FIGURE 2:**
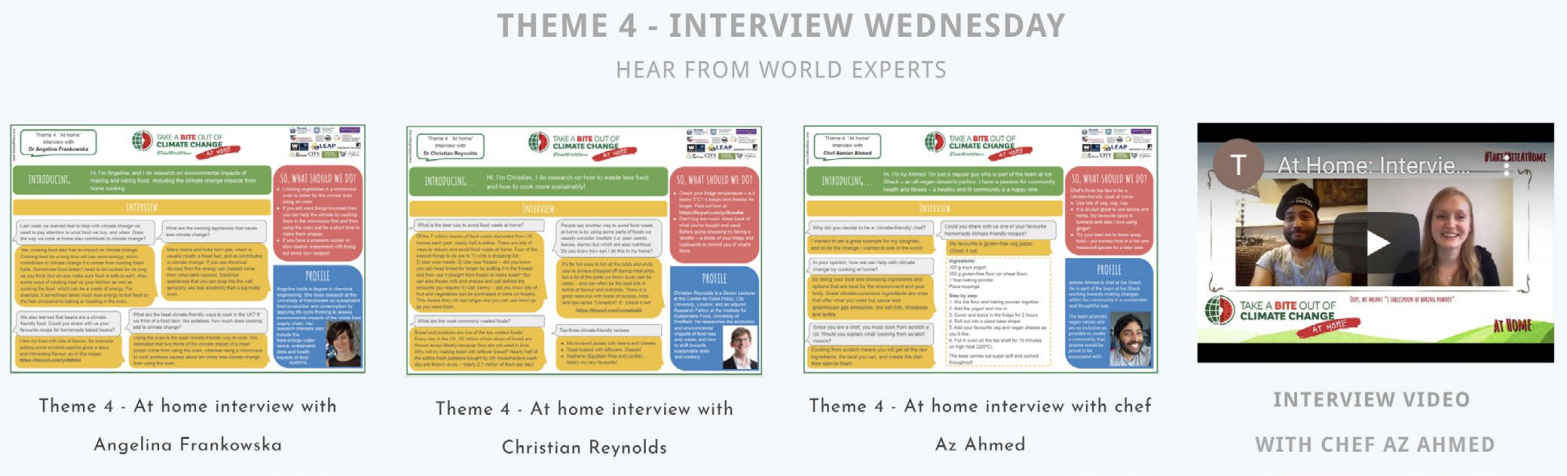
An example of the interview activity sheets and video interview [Colour figure can be viewed at wileyonlinelibrary.com]

Written interviews with three experts about each theme, asking some big‐picture questions as well as some top tips that schoolchildren and teachers could act on during lockdown. The interviews were made into PDF documents that could be downloaded or viewed via the *TakeABiteCC* website. These were made available 1 week in advance for teachers to plan their lessons (Figure 2). A video interview was available on the YouTube channel and website every Wednesday (for the recording of the interviews, we used the video conferencing platform Zoom).

#### Q&A Thursdays

Questions on each weekly topic collected via our hashtag, email and other informal communications. The chair asked these questions to between three and five experts who provided answers.

#### Showcase Fridays

The *TakeABiteCC* team and schoolteachers showcased work that participants sent in. Each week a winning participant's submission was chosen and a prize (a pack of Climate Food Flashcards; https://www.takeabitecc.org/flashcards.html) was posted to them. The winning submission was published on the *TakeABiteCC* website (with the winner's consent). This forum allowed children's work to be celebrated in a wider context and encouraged participants to ‘tune in’ to see whether their work was featured in this weekly celebration.

#### References and details

A PDF containing details of the worksheet calculations, references for facts cited, references of relevant scientific papers and websites, details of the interviews and other materials used was published on our website every Friday.

All materials produced were analysed by schoolteachers and proofread by a language and communications expert to ensure they were at the right difficulty level and understandable for our target audience.

Given the unfunded nature of the work, it was not possible to create different resources for different age groups. However, some activities included a ‘Bonus challenge’ and an ‘I want more’ resources section for children who wanted to learn more about the topic. In addition, we included a variety of activities to provide options for young and older children. For example, written activity worksheets followed by tutorial videos made by children to aid understanding for smaller children and parents; introductory videos to explain each theme and what children would learn from it; cheat sheets and detailed annotated references aimed at parents/carers and older children.

## PROJECT EVALUATION

**FIGURE 3:**
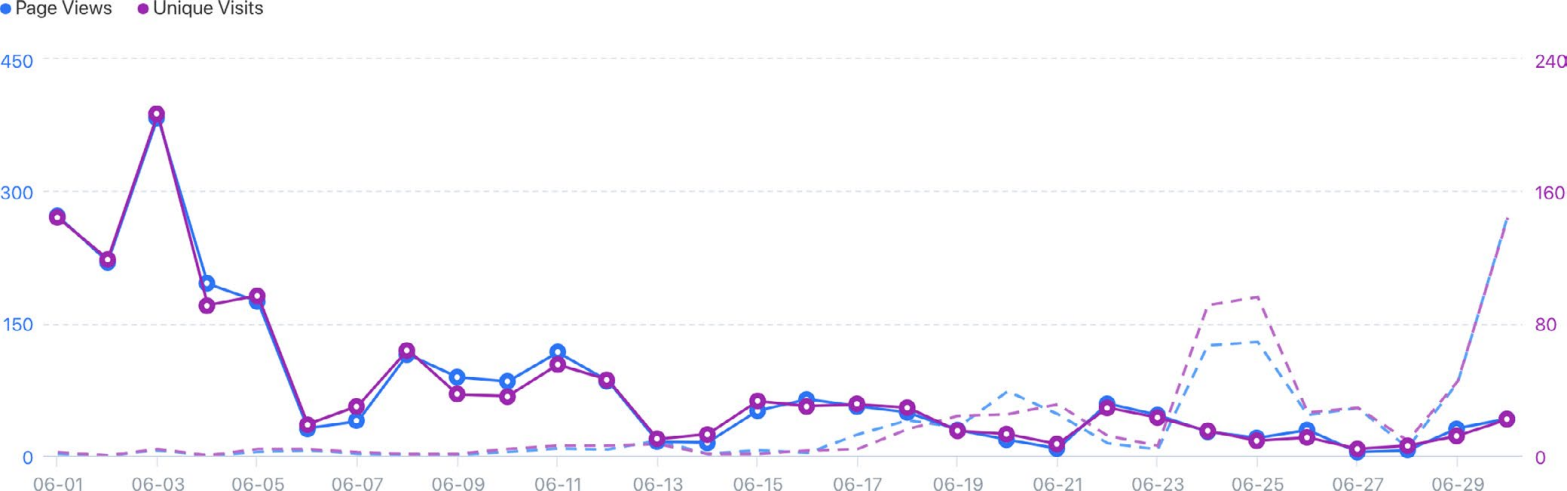
Website views for June (solid) and May (dashed) [Colour figure can be viewed at wileyonlinelibrary.com]

Twenty‐two videos were produced, with more than five and a half hours of content (337 min). This attracted a small but dedicated audience, with 24 subscribers, and (at least) 960 views over the course of the month (due to some videos having to be re‐uploaded as a result of editing errors this number of video views does not reflect the true view rates). Among the proposed activities, the most popular were those related to Themes 1 and 2 ‘From Planet to Plate’ and ‘At the Farm’, respectively. The introductory video ‘Go Monday’ of Theme 1 had 439 views on our YouTube channel, followed by the ‘Work‐it‐out Tuesday’ activity worksheet with 159 views, and 137 views for the ‘Interview Wednesday’ video. The activity worksheet of Theme 2 had 136 views. Figure 3 presents the website views for 1st June to 30th June (solid) and 1st May to 31st May (dashed). This shows a spike to ~390 page views per day at the start of June (solid, ~06 views) and another the week before when our materials were sent to schools in order to plan the lessons ahead (as requested by school headteachers) and also when linked from the *Great Science Share for Schools* – a campaign that invites schoolchildren aged 5–14 years to share their students’ scientific questions and investigations, in order to raise interest in science with schools and communities. The number of views trailed off towards the end of June. Although these numbers are not huge, they show an increased number accessing the website when compared with the previous month and represent a larger audience than would be achieved in a typical school classroom setting. Additionally, the style of materials means they can easily be re‐used by both teachers and families at home.

We received strong positive feedback over social media and email, with some brilliant work being sent in each week by schoolchildren who engaged (and went beyond) the materials. Inspired by our activities, a group of primary schoolchildren printed out our flashcards, from the Activity Worksheet Theme 4, in A4 size to play a game where they guessed the GHGE of different foods. The activity was filmed by their teacher and sent to us. Many drew on the previous week's activities to enhance the following week's activities. For example, one student sent in a worksheet comparing the GHGE impacts of taking a shower versus their lunch versus driving to the shops – separate activities from different weeks. Feedback from teachers about the resources includes the following:

- The material you've provided is amazing and extremely thought provoking; it will make the students think!’ – Secondary School Biology Teacher
- Love this! I'm organising an activity week at school and was using the countryside classroom resources and this links in perfectly especially for our older kids!’ – KS2/3 Geography Teacher

Schoolchildren also shared their experiences of changes they made as a result of *TakeABiteAtHome*. These included growing vegetables, trying new foods, changing transport or shopping habits, and noticing new (local) foods in their environment. Similarly, small changes in daily behaviour towards climate change due to knowledge acquired, such as discussions with parents and friends about the subject, recycling, turning off lights, taking shorter showers and unplugging unused electronics, were identified by Flora et al., (2014) in high school students attending entertainment‐education presentations on climate science.

## IMPLICATIONS FOR RESEARCH AND PRACTICE

Before, during and after the *TakeABiteAtHome* month of activities, we had meetings with stakeholders from different sectors (teachers, public engagement experts, mental health specialists, etc.) on the mode of delivery and the potential impact of the activity on teachers, students (including their families) and school communities. This section discusses how these public engagement materials could be developed, looking at the quality of the content, the delivery method and the impact of the activities on the participants.

### Power dynamics of children, diet and climate change

Throughout the *TakeABiteAtHome* month of activities, we used our materials (*e*.*g*. Work‐it‐out Tuesdays’ worksheet) to suggest different actions that individuals could engage with to reduce their climate impact. We also encouraged a two‐way process of participation and interaction generating mutual benefit. This was done with two days of the activities (Q&A and Showcase Fridays) in which we explored the participatory nature of public engagement while also providing opportunities for creativity. The final Q&A session was with policy and education influencers in the UK, providing inspiration and discussion about how children could influence food producers and governments. However, a question for the Theme 4 Q&A panel highlighted that these actions happen within a family unit, and the power to enact these changes may not sit with children but with the families’ decision‐maker(s), even though children play an important role in families’ decision‐making (Lawson et al., 2019). Due to this need for wider family action and communication, an additional information sheet was developed with bespoke stories from Ro Randall, a psychotherapist with experience in helping children communicate with adults on climate action (and participant in the ‘At Home’ Q&A). This sheet provided tips on talking to family members about climate and dietary change from a child's perspective and used stories to help children understand when is a good time to approach adults and/or decision‐makers about climate and food issues. In retrospect, this use of stories, and additional content on how to engage family decision makers in changing diet was a crucial (and previously overlooked) part of the *TakeABiteAtHome* process. Future engagement must provide participants with support and advice so that they can approach their family members and wider community to discuss any issues raised.

### Mental health, diet and COVID‐19

At the start of the activities, a colleague expressed concern that influencing children to change their diet could lead to, or exacerbate, mental health issues around food. By encouraging children to think about their food choices and climate change, we were concerned that their anxiety related to eating or thinking about the future would increase. To address this issue the materials produced were analysed by Dr Chantal Basson (Lead Consultant, Pennine Care NHS Foundation Trust) and Ursula Arens (Member of the Sustainable Diets Specialist Group of the British Dietetic Association). Additionally, mental health and nutrition health warnings were placed on the *TakeABiteCC* website. The nutrition health warning advised people to take care, particularly if significantly changing their diets, and to check with a healthcare professional if there were any pre‐existing health or food issues. It mentioned that reducing intake of animal‐based foods can be done in a healthy way but may require planning around particular nutrients such as vitamin B12. This included advising contacting healthcare professionals and links to resources for childhood anxiety during the COVID‐19 lockdown. We also felt that empowering through knowledge and ideas for action could help manage anxieties, so we designed videos focused around empowering messages, with the communication style geared for children not only in terms of accessibility, but also tone. We would recommend any future engagement to map potential issues related to mental health and anxiety and develop a list of age‐ and audience‐appropriate empowering resources at the start of the project.

### Engaging the wider science, agriculture and food communities

*TakeABiteAtHome* ran concurrently with other online events including LEAF *Online Farm Sunday* (7 June 2020, #LOFS20, https://farmsunday.org/online‐farm‐sunday), where farms across the UK posted videos about life on a farm to enable the wider community to learn and experience what happens on a farm, and *Great Science Share* (16 June 2020, #GreatSciShare, https://www.greatscienceshare.org/), an annual campaign for young people to share their scientific questions, as previously mentioned. Individuals involved in these events co‐promoted and retweeted each hashtag. Our engagement results show a spike in views when our project was linked with the *Great Science Share*. However, one tension that emerged was that livestock and dairy farmers from *Online Farm Sunday* took issue with the broad messages to reduce consumption of animal products, highlighting their carbon footprint mitigation actions over Twitter (a forum with a 13+‐year‐old minimum age requirement), which is away from our main audience of schoolchildren. This provided us with an opportunity for engagement with the wider community to discuss these issues and allowed us in Q&A videos to raise these issues in language appropriate to our audience, turning technical scientific concepts into approachable discussions. Several studies have reported the use of discussions with worksheets (Monroe et al., 2019; Theobald et al., 2015; Oluk & Özalp, 2007; Reinfried, Aeschbacher & Rottermann 2012; Sellmann & Bogner, 2013b) as a positive, engaging strategy, in part due to learners sharing ideas and observations, and coming to new understandings as a result. To ensure an appropriate education on climate change, educators targeted in those studies tackled climate misconceptions in many ways, such as simplifying information and through constructive reflection and discussions.

Future engagement activities need to be aware of the wider ecosystem of activities that are taking place to allow for synergy between activities, so that if issues or conflicts arise these can be used as moments of engagement and further teaching.

It should also be noted that significant events occurred during *TakeABiteAtHome* that might have impacted its reach on social media. The Black Lives Matter (BLM) social media presence during June 2020 was rightfully large, with all people and institutions focusing their communication on it.

### Unfunded and homemade nature of this programme

*TakeABiteAtHome* was carried out entirely unfunded, based on materials developed for the Royal Society Summer Science Exhibition 2019 (funded by STFC Food Network+and the HEFCE Catalyst‐funded N8 AgriFood Resilience Programme, matched funding from the N8 group of universities) and ideas arising from workshops funded by the Wellcome Trust previous to March 2020. However, due to its unfunded nature, we had no external requirements on content, delivery method or style, giving us total freedom and a much faster response time to develop new material. Its planning, development and sharing were made possible through free and/or accessible platforms (such as PowerPoint, Zoom, YouTube, social media), in addition to the *TakeABiteCC* website. Also, the team involved in the development of these materials (researchers, guests and collaborators) worked as volunteers. Despite the difficulties in developing educational materials without funding, even though this project would have achieved better reach with funding, it has shown that it is possible to develop free, evidence‐based educational content in a short time and with reduced resources with a group of dedicated volunteers. In this sense, our experiences can be used as an example for developing educational materials for other food‐related topics, facilitating the dissemination of science through the school community. However, this was largely possible due to unforeseen availability as a result of changes of schedule due to lockdown (cancelled examinations and travel), and the culture of spontaneous homebrewed content that arose during the first lockdown period. Furthermore, the use of remote working during a socially challenging time was an important glue that held the core team together, despite many members having never met previously.

### Food system, STEAM and diversity

*TakeABiteAtHome* offers a brief snapshot of the complexities of the food system and how different disciplines are required to make food production and consumption more sustainable and climate change friendly. Our materials and resources show: (1) how classroom activities can help to teach about climate change providing a series of fun and science‐based materials such as worksheets, references and other supporting resources, as well as how they have been already used and adapted at home and in schools (Showcase Friday); (2) promote and encourage STEAM (science, technology, engineering, arts and mathematics), social sciences and vocational disciplines through written and recording interviews (Interview Wednesday) and Q&A sessions (Thursday) with a diverse range of experts. Rudd et al. (2020) produced a public engagement study, which showed that multidisciplinary approaches towards STEAM and the food systems provide students with an opportunity to explore themes around climate change, which was reflected in the digital fictions written by the students. This approach was implicitly reinforced every day as *TakeABiteAtHome* itself consists of cosmologists, physicists, biologists, engineers, mathematicians, nutritionists, psychologists and teachers. Our online approach allowed us to also open up to international contributors and content (Brazil, India, the US, etc.)

### Part of an ongoing journey

This project corroborates what is being prioritised in the international educational agenda, such as the ideas presented on the UNESCO’s *Education in a post*‐*COVID world* report (International Commission on the Futures of Education, 2020), which calls for ‘the mobilisation and participation of all in shaping the futures of education’. A practical contribution of *TakeABiteAtHome* is that it provides much needed collaborative actions built with the school community (teachers, children and families), acting as a vehicle to promote the participation of the school community in the co‐construction of food and climate change understanding, and the possible/desired changes. As a strategy to continue offering opportunities to other school communities to incorporate and maintain food and climate change lessons, these free evidence‐based educational materials will remain available on our website, allowing their use in the future and benefiting, therefore, other audiences.

There is an increasing appetite from young people to learn more about climate change at school. Through these tools, we have demonstrated the importance and potential to include food as part of that discussion, tying in with existing curriculum elements including healthy food choices. We have brought together creative and quantitative elements to learn about and illustrate the cumulative and comparative impact of different decisions. To continue to provide up‐to‐date and relevant curriculum support, we also need to connect with other bodies that are looking to have an impact with children through projects such as the World Wide Fund for Nature ‘Sustainable Schools’ programme, Eco‐Schools, Healthy Schools programme and others. These projects aim to put ‘Green’ education and sustainability at the heart of school life and would therefore offer the ideal partners to develop additional food‐based climate change resources. Schools already struggle to develop food technology in a meaningful way and extending support through working with specialists with real‐world working knowledge would greatly improve children's experiences of this and other STEAM subjects.

To bring this topic to a large number of schools in a practical way in the future, the materials should be comprehensively mapped to the existing curricula and extended to add more examples that tie‐in with existing elements. Due to the finite amount of time during lockdown, the materials are aimed at a broad age range of 7‐14 year‐olds. However, for more effective learning the materials should be tailored to narrower age and/or ability ranges. Furthermore, the materials should be beta‐tested with teachers and students to improve them further – ideally leading to co‐development of additional materials responding to students’ interests. Another step for this project will be to run with sufficient funds and resources that allow us to measure the impact of the programme on knowledge, attitudes and behaviour. An ambition might be to bring the project to a more diverse audience, for instance families that are not already part of the ‘climate aware’ middle class. We are proud of the diversity of the people featured in the videos, but we would like to extend this further, perhaps including interviews with experts from around the world, covering a wider range of ethnicity and other diversity. Finally, due to the practicality of Internet speeds, there are several glitches in quality in the videos that would ideally be ironed out.

As education continues to adapt in order to address the growing concerns of the 21st century child, more and more content needs to be added to an already full curriculum. *TakeABiteAtHome* daily videos and challenges managed to create opportunities that are easily adaptable to address the basics of Maths and Science objectives without adding in extra pressure. Although this time of reduced face‐to‐face learning gives teachers a new freedom of delivery, it also highlights the issues facing coverage of missed skills. Through relating data and calculation skills to climate change, more than one area of the curriculum can be addressed which also allows for real‐world contexts to translate learning into practical understanding. The activities produced were fun, engaging and could be completed independently by children; however, most were completed alongside adults, which allowed for conversations around climate impact and personal situations to be explored. Children, therefore, have gained an invaluable amount of information on what impact they have on their world as well as ways of making positive changes without the pressure of guilt. Although there are still conversations to be had around working alongside other organisations (farming unions, etc.), the basis of a relevant and forward thinking curricula which addresses STEAM skills has already had an impact, and for the schools involved, conversations are already underway on developing these topic areas further with more children across the key stages.

## CONCLUSION

Using online learning platforms is likely to become more embedded as we continue the 2020/2021 school year. *TakeABiteAtHome* gave children the opportunity to ask questions and get those questions addressed by experts online. It would be expected to give food impact on climate change a priority to each individual's learning. Children seeing their work recognised and praised encourages them to continue to hold these areas as important and has meant that these necessary conversations about food impact are being had beyond the classroom. Although we recognise that not all children are in a position to make significant immediate changes to the food consumed by their household, they are in a position to influence others in the impact of the food choices being made as shown previously in Lawson et al., (2019). This led to schoolchildren asking to grow their own vegetables and discussing the origins of fruit and vegetables and the impact of transport on eating habits. The more conversations that children have about food production and consumption with all adults around them – the sooner adult decisions will be questioned and hopefully changed – placing more priority on how eating habits can be changed for the good of the planet and all of our futures.

## Conflict of interest

All authors have no conflicts of interest to disclose.
